# Impact of Digital Interventions in Occupational Health Care: A Systematic Review

**DOI:** 10.1016/j.mcpdig.2025.100216

**Published:** 2025-03-18

**Authors:** Mirjam M. Jern-Matintupa, Anita M. Riipinen, Merja K. Laine

**Affiliations:** a Department of General Practice and Primary Health Care, University of Helsinki and Helsinki University Hospital, Helsinki, Finland; b Terveystalo, Helsinki, Finland; c Folkhälsan Research Center, Helsinki, Finland

## Abstract

**Objective:**

To assess the existing body of evidence and impact of digital interventions on occupational health care.

**Methods:**

The search strategy and review process were conducted in accordance with the PRISMA guidelines. The search was carried out during a period from January 1, 2013 to June 5, 2023, using the SCOPUS and Ovid Medline databases. After the identification of the relevant records, screening was conducted in 3 stages, following specific predetermined inclusion and exclusion criteria. A data-extraction model was created on the basis of the aim of the review. The quality of the selected studies was evaluated using the Effective Public Health Practice framework. Owing to the heterogeneity of the outcome measures, we used narrative synthesis to summarize the findings.

**Results:**

We identified 382 records in SCOPUS and 441 in Ovid Medline. We selected 54 studies to be included in the evidence synthesis. The health targets of the interventions varied widely, but we identified 2 main focus areas: sedentary behavior (n=17, 32%) and mental health (n=14, 26%). Even when the studies had the same health target, the outcomes and chosen measures varied widely. Given the considerable effect of the primary outcome, mental health appears to be a good target for digital interventions. Online training and computer software could be especially effective.

**Conclusion:**

The potential positive impact of digital interventions on mental health, especially online training, should be leveraged by health care professionals and providers. In order to provide more specific recommendations for health care professionals, occupational health care researchers should strive for consensus on outcome measures.

**Article Highlights**

• Digital interventions should be considered, not only for mental health promotion but also for healthy lifestyle and cardiovascular health.
• Online training appears to be suitable for addressing mental health issues such as burnout and depression in the workplace, and although they require initial investment, such interventions are subsequently quite easily scalable.
• Given that mental health is now a major global concern, especially among younger adults, all tools that can help reduce the mental health-related disease burden are extremely important, from both societal and employer perspectives.
• A consensus on specific outcome measures for evaluating the impact of digital health in occupational health care is essential for obtaining easily comparable results and providing more detailed advice for health care professionals and policy makers.

Workplace wellbeing is becoming increasingly important, emphasizing preventive measures and to move beyond traditional boundaries. The current key focuses are promoting mental health, preventing overwork, managing the labor force, and enhancing safety. Improved occupational health care benefits employees, employers, and the broader community, creating a transition from worker health to citizen health.^1^, ^2^, ^3^, ^4^, ^5^ Digitalization and globalization are also impacting our work practices and preferences, and this new trend, known as work 4.0, is accompanied by health implications such as increased stress, musculoskeletal problems, and mental health issues.^3^^,^^6^

The World Health Organization defines digital health as “a broad umbrella term encompassing eHealth (which includes mHealth), as well as emerging areas, such as the use of advanced computing sciences in ‘big data’, genomics and artificial intelligence.”^7^ eHealth in turn is defined as “the use of information and communications technology in support of health and health-related fields” and mHealth as “the use of mobile wireless technologies for health.” mHealth falls under the concept eHealth.^7^

Digital interventions are increasingly being used in response to the current needs and trends in occupational health care, yet there is still much room for improvement compared with their use in data-driven fields.^2^ Digital interventions are cost effective and scalable, which means they show potential for achieving health benefits. An aspect to consider in the use of digital interventions is engaging patients in the use of new digital tools. Health care professionals, especially physicians, play a key role in this because a recommendation from a physician can be vital in promoting this needed engagement. However, health care professionals require solid scientific evidence before they are willing to recommend a new tool.^8^, ^9^, ^10^, ^11^, ^12^, ^13^, ^14^

Despite the vast potential of digital interventions for addressing occupational health care needs, we still need to know more about which digital interventions to focus on in order to improve key prevention targets in occupational health care, which digital interventions to develop, and how to do this. Thus, the aim of this systematic review was to assess the existing body of evidence on the overall impact of digital interventions in occupational health care, in both experimental and real-world settings.

## Methods

### Search Strategy and Selection Criteria

This study was a systematic review. We included all types of digital interventions that aligned with the definition given earlier: any digital, mobile, or wireless technology used to promote occupational health objectives. This encompasses interventions such as smartphone applications, wearables, chat platforms, messaging services, virtual appointments, and patient portals. In order to capture as comprehensive a view of the existing evidence as possible, we imposed no restrictions in terms of occupation or health targets. Moreover, as long as the study focused on the digital aspect, the intervention did not have to be exclusively digital; it could also encompass other aspects.

During the identification of relevant record duplicates, we excluded articles published before 2013 and not translated into English, as well as book chapters. We also excluded the following types of texts: review articles, editorial pieces, letters, and opinions. Studies that did not focus on evaluating the impact of a digital intervention or whose therapy area was not occupational health care were also excluded. Supplemental Appendix 1 (available online at https://www.mcpdigitalhealth.org/) presents the reasons for excluding each study at the full-text stage.

The search strategy and review process were conducted in accordance with the PRISMA guidelines.^15^ We used the SCOPUS and Ovid Medline databases to search for results; the main search was conducted on June 5, 2023. Based on the mapping concept and PICO methodology^16^ for answering research questions, we combined the following terms for the search: *digital health interventions*, *mhealth*, *mobile health*, *telemedicine*, *health application*, *e-health*, OR *health-IT* with *occupational health care* AND *occupational health*, AND further with *impact*, *assessment*, *improved health*, *improved outcomes*, and *value*. Supplemental Appendix 2 (available online at https://www.mcpdigitalhealth.org/) presents more in-depth details on the search strategy. All records were screened independently by 2 reviewers (M.M.J.-M and A.M.R. or M.K.L.), and when opinions differed, all 3 reviewers evaluated the record in question and discussed whether it should be included or excluded. The full text of the remaining studies was assessed independently by 2 of the authors (M.M.J.-M. and A.M.R. or M.K.L.), using the same procedure as used for the screening. When the full text was not immediately available, the corresponding authors of the articles in question were contacted (3 corresponding authors were contacted, of who 2 responded and made the articles available for this review). M.M.J.-M. built a data-extraction model in Excel which the other reviewers tested (Table 1). After approval, data were extracted from the remaining studies. Data on publication details were automatically extracted via Zotero, whereas all other data were manually retrieved by the reviewers.

**Table 1:** Data-Extraction Model Variables

Publication details	Study details	Population	Intervention	Results	Conclusion	Bias
Title	Start date	Type of work^a^	Type of intervention^b^	Time point for follow-up	Conclusion	Evaluation of bias^c^
Publication year	End date	Work classification	Health target^d^	Timepoint for follow-up (weeks)		
Author	Year conducted	Inclusion criteria	Duration of intervention	Primary outcome^e^		
Publication title	Country	Exclusion criteria		Measure (primary outcome)		
ISSN	Study design	Sample size		Outcome (primary)		
DOI	Study design	Recruitment process		Secondary outcomes		
	Objective	Intervention group: age		Outcome (secondary)		
	Retention rate^f^	Intervention group: sex		Measure (secondary outcome)		
	Start date	Control (yes or no)		Significant or not, primary outcome		
	End date	Control group age		Significant or not, primary outcome		
		Control group sex				

DOI, digital object identifier; ISSN, international standard serial number.

a Type of work was classified as either blue-collar (manual labor) or white-collar (office work, admin work, or similar) work.

b Type of intervention was classified as smartphone applications, online interventions (delivered via the internet), computer software (local delivery), wearables, blended (digital component paired with nondigital intervention), online training (longer interventions with clear educational component), or a combination of any of the mentioned interventions (4 of the blended interventions contained a smartphone application component).

c Bias was evaluated by the Effective Public Health Practice Project (EPHPP) and reported as quality of study: weak, moderate, or strong.

d Health target, that is, the target of the intervention was classified into sedentary behavior; mental health; healthy lifestyle; cardiovascular diseases; diabetes mellitus; complaints of the arm, neck, and shoulder (CANS); sickness absence; access to care; or process or a combination of the mentioned targets.

e If not stated clearly in the study, primary outcome was assumed to be the first outcome measured.

f Retention rate was calculated as the population at the end of the study divided by the population at the start of the study.

### Data Analysis

Owing to the heterogeneity of the outcome measures, we used a narrative synthesis to summarize the findings. The significance was determined according to a *P*-value of <.05, CIs, odds ratios, or similar metrics. Three studies used a mixed-method or qualitative approach, which meant they did not report the statistical significance of their primary outcomes.

To evaluate bias and the quality of the studies, we used the Effective Public Health Practice Project model. This model takes several categories such as study design, population selection, and confounders into consideration. All categories are graded, and a final classification is calculated.^17^ The choice of the evaluation tool was based on the need to support all types of studies and on its previous successful evaluations of digital interventions.^10^ Supplemental Appendix 4 (available online at https://www.mcpdigitalhealth.org/) shows the final classification into weak, moderate, or strong.

## Results

In total, 382 records were identified in SCOPUS and 441 in Ovid Medline in June 2023, which had been published between January 2013 and June 2023. After screening and eligibility evaluation, 59 studies remained. After we applied the data-extraction model, the final number of studies selected for the evidence synthesis was 54 (Figure, Supplemental Appendixes 2 and 3, available online at https://www.mcpdigitalhealth.org/).

**Figure:**
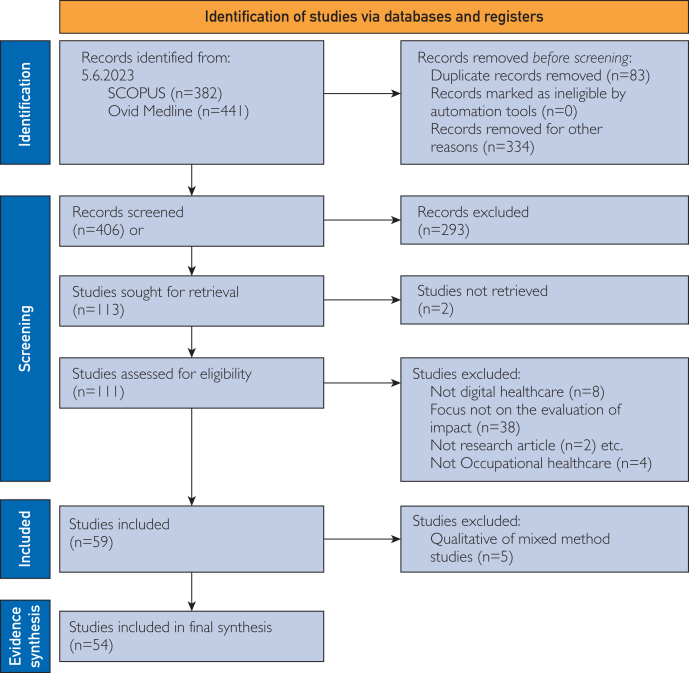
PRISMA flow chart of screening of records on impact of digital interventions in occupational health care.^15^

### Main Characteristics

Table 2^11^^,^^18^, ^19^, ^20^, ^21^, ^22^, ^23^, ^24^, ^25^, ^26^, ^27^, ^28^, ^29^, ^30^, ^31^, ^32^, ^33^, ^34^, ^35^, ^36^, ^37^, ^38^, ^39^, ^40^, ^41^, ^42^, ^43^, ^44^, ^45^, ^46^, ^47^, ^48^, ^49^, ^50^, ^51^, ^52^, ^53^, ^54^, ^55^, ^56^, ^57^, ^58^, ^59^, ^60^, ^61^, ^62^, ^63^, ^64^, ^65^, ^66^, ^67^, ^68^, ^69^, ^70^ presents the main characteristics of all studies (N=54) included in the evidence synthesis. Of these studies, 22 (41%) focused on white-collar workers, 7 (13%) on blue-collar workers, and 25 (46%) examined a mix of blue-collar and white-collar workers. Further analysis revealed that the health care sector (n=9, 19%) and office workers (n=13, 24%) were common study populations. Thirty studies (56%) were randomized controlled trials (RCTs), making this the most common study type. Thirty-two studies (59%) had control groups. With respect to the types of digital interventions used, 24 studies (44%) used smartphone applications, either alone or as part of a multicomponent intervention. The health targets of the interventions varied widely, but we identified 2 primary focus areas: sedentary behavior (n=17, 32%) and mental health (n=14, 26%). Additionally, 2 studies (3.7%) addressed a combined outcome of mental health and sedentary behavior.

**Table 2:** Main Characteristics of Studies Included

Reference, year	Country	Study design	Work classification	Sample size	Intervention	Target of intervention	Primary outcome	Significance of primary indicator
Carr and Kevitt,^18^ 2023	Ireland	Cross-sectional	Mixed	73	Telecare	Access to care	User satisfaction	NA
Willman,^19^ 2023	United Kingdom	Mixed-method evaluation	White-collar	135	Telecare	Access to care	NA	NA
Hutting et al,^20^ 2015	Netherlands	RCT	Mixed	123	Blended intervention	CANS	Disabilities of the arm, shoulder and hand questionnaire score	Not significant
Kouwenhoven-Pasmooij et al,^21^ 2018	Netherlands	RCT	Mixed	493	Online training/intervention	Cardiovascular health	Self-rated health	Significant
Ryu et al,^22^ 2021	Korea	Intervention study	White-collar	87	Application	Cardiovascular health	Cardiovascular-related health status	Significant
Simons et al,^23^ 2017	Netherlands	RCT	White-collar	116	Blended intervention	Cardiovascular health	Cardiovascular health, total cholesterol, low-density lipoprotein, high-density lipoprotein, blood pressure, blood glucose, and glycated hemoglobin	Significant
Widmer et al,^24^ 2014	United States	Cohort	Mixed	152	Online training/intervention	Cardiovascular health	Framingham Risk Score	Significant
Widmer et al,^25^ 2016	United States	Cohort	Mixed	30,974	Application, online training/intervention	Cardiovascular health	Weight, waist circumference, body mass index, blood pressure, lipids, and glucose at 12 mo	Significant
Lavaysse et al,^26^ 2022	United States	RCT	Mixed	125	Application, wearable, messages	Diabetes	Absenteeism and presenteeism	Not significant
Nagata et al,^27^ 2022	Japan	RCT	Mixed	103	Application, wearable, e-mail	Diabetes	Glycated hemoglobin	Significant
Nundy et al,^28^ 2014	Chicago, United States	Quasi-experimental	Mixed	74	Messages	Diabetes	Glycated hemoglobin, lipid profile, body mass index, and blood pressure	Significant
Röhling et al,^29^ 2020	Germany	RCT	Mixed	30	Blended intervention	Diabetes	Difference in weight reduction	Significant
Balk-Møller et al,^30^ 2017	Denmark	RCT	Mixed	566	Blended intervention	Healthy lifestyle	Changes in body weight	Significant
Johnson et al,^31^ 2021	United States	Intervention study	Mixed	296	Telecare	Healthy lifestyle	Weight loss percent	Significant
Kempf et al,^32^ 2019	Germany	RCT	White-collar	104	Telecare	Healthy lifestyle	Body weight reduction at 12 mo	Significant
Park et al,^11^ 2022	South Korea	Prospective observational study	Mixed	1171	Application	Healthy lifestyle	Changes in systolic and diastolic blood pressure, body weight, body mass index, waist, circumference, fasting blood glucose, triglyceride, and high-density lipoprotein cholesterol levels	Significant
Wipfli et al,^33^ 2019	United States	Cohort	Blue-collar	134	Online training/intervention	Healthy lifestyle	Body weight, fruit and vegetable consumption, and physical activity	Significant
Bolier et al,^34^ 2014	Netherlands	RCT	White-collar	1140	Online training/intervention	Mental health	Positive mental health	Significant
Comtois et al,^35^ 2022	United States	RCT	Mixed	1356	Application	Mental health	Anxiety and suicidal behavior	Significant
Costa et al,^36^ 2022	United States	Cohort	Mixed	7785	Application, wearable	Mental health	Mean change in depression	Not significant
De Kock et al,^37^ 2022	United Kingdom	RCT	Mixed	169	Application	Mental health	Mental wellbeing and anxiety	Significant
Ebert et al,^38^ 2018	Germany	CBA	White-collar	264	Online training/intervention	Mental health	No. of participants who achieved symptom-free status	Significant
Gayed et al,^39^ 2019	Australia	RCT	White-collar	210	Online training/intervention	Mental health	Change in managers’ self-reported confidence in creating a mentally healthy workplace in which the mental health needs of their employees are appropriately supported	Significant
Gwain et al,^40^ 2022	United States	Cohort	White-collar	43	Messages	Mental health	Prevalence of depressive symptoms	Significant
Jukic et al,^41^ 2020	Slovenia	Intervention study	White-collar	17	Application	Mental health	Participants’ cardiovascular fitness levels	Not significant
Lokman et al,^42^ 2017	Netherlands	RCT	Mixed	220	Online training/intervention, e-mails	Mental health	Health care use and absenteeism from work	Significant
Meyer et al,^43^ 2018	Australia	Mixed-method evaluation	Mixed	178,350	Application, wearable, online training	Mental health	Psychological wellbeingwell	Significant
Michelsen and Kjellgren,^44^ 2022	Sweden and United Kingdom	Intervention study	Mixed	50	Online training/intervention	Mental health	Risk of burnout	Significant
Sasaki et al,^45^ 2021	Vietnam	RCT	Blue-collar	962	Application	Mental health	Work engagement	Not significant
Umanodan et al,^46^ 2014	Japan	RCT	Blue-collar	266	Computer software	Mental health	Psychological distress, work engagement, and work performance	Significant
Volker et al,^47^ 2015	Netherlands	RCT	Mixed	220	Online training/intervention	Mental health	Duration until first return to work	Significant
Atkins et al,^48^ 2020	Finland	RCT	Mixed	26,804	Blended intervention	Process	Education of mean number of medium-term (4-14 calendar days) sickness absences	Not significant
Chen et al,^49^ 2019	China	RCT	Blue-collar	1211	Blended intervention	Process	Self-reported appropriate use of respiratory protective equipment	Significant
Boerema et al,^50^ 2019	Netherlands	Intervention study	White-collar	15	Application, wearable	Sedentary behavior	Physical activity, total sedentary time, and No. of sedentary spells	Not significant
Bort-Roig, et al,^51^ 2020	Spain	RCT	White-collar	141	Application	Sedentary behavior	Total sitting time, sedentary spells, and breaks	Not significant
Cooley et al,^52^ 2014	Australia	Qualitative evaluation	Mixed	46	Computer software	Sedentary behavior	Microsystem, mesosystem, and exosystems of Bronfenbrenner (1992) model	Not applicable
Gilson et al,^53^ 2017	Australia	Intervention study	Blue-collar	44	Blended intervention	Sedentary behavior	Physical activity	Not significant
Huang et al,^54^ 2023	Taiwan	RCT	White-collar	223	Application, online training, messages	Sedentary behavior	Physical activity measured using metabolic equivalents	Not significant
Judice et al,^55^ 2015	Portugal	RCT	White-collar	10	Computer software	Sedentary behavior	Sitting time and standing time, sit/stand transitions	Significant
Kouwenhoven-Pasmooij et al,^56^ 2017	Netherlands	Intervention study	Blue-collar	51	Blended intervention	Sedentary behavior	Average physical activity per day	Significant
Lau and Faulkner,^57^ 2019	Canada	Intervention study	Mixed	843	Blended intervention	Sedentary behavior	Daily steps	Not significant
Lee et al,^58^ 2019	South Korea	Quasi-experimental	Mixed	79	Blended intervention	Sedentary behavior	Daily walking steps	Significant
Lennefer et al,^59^ 2020	Germany	Intervention study	White-collar	121	Application, online training/intervention	Sedentary behavior	No. of steps, job control, self-efficacy, emotional strain, and negative effect	Significant
MacDonald et al,^60^ 2020	United Kingdom	Mixed-method evaluation	White-collar	80	Application	Sedentary behavior	Streaks in sedentary behavior at work and total sedentary behavior at work	Not significant
Mainsbridge et al,^61^ 2014	Australia	RCT	Mixed	29	Computer software	Sedentary behavior	Mean arterial pressure	Significant
Mainsbridge et al,^62^ 2018	Australia	Cohort	White-collar	228	Computer software	Sedentary behavior	Mean arterial pressure	Significant
Maylor et al,^63^ 2018	United Kingdom	RCT	White-collar	89	Blended intervention	Sedentary behavior	Workplace sitting time	Not significant
Morris et al,^64^ 2020	Australia	RCT	White-collar	56	Application	Sedentary behavior	Behavioral outcomes (worktime sitting, standing, stepping, prolonged sitting, and physical activity)	Significant
Pedersen et al,^65^ 2014	Australia	RCT	White-collar	34	Computer software	Sedentary behavior	Workplace daily energy expenditure	Significant
Thogersen-Ntoumani et al,^66^ 2020	Australia	RCT	White-collar	97	Blended intervention	Sedentary behavior	Steps, standing, and sitting	Not significant
Haile et al,^67^ 2020	United Kingdom	Intervention study	White-collar	41	Application	Sedentary behavior and mental health	Sitting and physical activity	Significant
Muniswamy et al,^68^ 2022	India	RCT	White-collar	64	Online training/intervention, social media, telecare	Sedentary behavior and mental health	Physical health	Not significant
Notenbomer et al,^69^ 2018	Netherlands	RCT	Mixed	82	Online training/intervention	Sickness absence	No. of register-based sickness absence episodes	Not significant
Van Schaaijk et al,^70^ 2019	Netherlands	RCT	Blue-collar	124	Application	Work ability	Work ability, vitality, work-related fatigue	Not significant

Statistically significant: *P*<.05.

CBA, cost-benefit analysis; CANS, complaints of the arm, neck and shoulder; NA, not applicable; RCT, randomized controlled trial.

Ten studies were conducted in the Netherlands^20^^,^^21^^,^^23^^,^^34^^,^^42^^,^^47^^,^^50^^,^^56^^,^^69^^,^^70^; 9 each in Australia^39^^,^^43^^,^^52^^,^^53^^,^^61^^,^^62^^,^^64^, ^65^, ^66^ and in the United States of America^24^, ^25^, ^26^^,^^28^^,^^31^^,^^33^^,^^35^^,^^36^^,^^40^; 5 in the United Kingdom^19^^,^^37^^,^^60^^,^^63^^,^^67^; 4 in Germany^29^^,^^32^^,^^38^^,^^59^; 3 in South Korea^11^^,^^22^^,^^58^; and 2 in Japan.^27^^,^^46^ Finally, Canada, China, Denmark, Finland, India, Ireland, Portugal, Slovenia, Spain, Sweden, Taiwan, and Vietnam^18^^,^^30^^,^^41^^,^^44^^,^^45^^,^^48^^,^^49^^,^^51^^,^^54^^,^^55^^,^^57^^,^^68^ all had 1 study each.

### Significance of Primary Outcome

Regarding the significance of the primary indicator, 51 studies were included in the analysis. Three studies^18^^,^^19^^,^^52^ did not report statistical significance of the primary outcome and were thus excluded from this part of the analysis. A significant effect of the primary outcome was observed for 32 of the 51 (63%) studies. Table 3 presents the significance of the primary outcome by target and Table 4 by digital intervention.

**Table 3:** Significance of Primary Outcome Reported by Health Target

Health target	Significant	Not significant	Total
Complaints of the arm, neck and shoulder	0	1	1
Cardiovascular health	5	0	5
Diabetes	3	1	4
Healthy lifestyle	5	0	5
Mental health	10	4	14
Process measure	1	1	2
Sedentary behavior	8	8	16
Sedentary behavior and mental health	1	1	2
Sickness absence	0	1	1
Work ability	0	1	1
Total	33	18	51

**Table 4:** Significance of Primary Outcome by Digital Intervention

Type classification	Significant	Not significant	Total
Application	5	6	11
Application, messages, online training	0	1	1
Application, online training/intervention	2	0	2
Application, wearable	0	2	2
Application, wearable, e-mails	1	0	1
Application, wearable, messages	0	1	1
Application, wearable, online training	1	0	1
Blended intervention	7	5	12
Computer software	5	0	5
Messages	2	0	2
Online training/intervention	7	2	9
Online training/intervention, e-mails	1	0	1
Online training/intervention, social media, telecare	0	1	1
Telecare	2	0	2
Total	33	18	51

As regards sedentary behavior, 8 of the 17 studies (47%) reported a significant positive effect.^55^^,^^56^^,^^58^^,^^59^^,^^61^^,^^62^^,^^64^^,^^65^ Smartphone applications were used in 7 of the 17 studies, 3 (43%)^59^^,^^64^^,^^67^ of which reported a significant effect.

A more detailed examination of mental health revealed that 11 of the 14 studies (79%) reported a significant positive effect.^34^^,^^35^^,^^37^, ^38^, ^39^, ^40^^,^^42^, ^43^, ^44^^,^^46^^,^^47^ Of these, all 6 studies^34^^,^^39^^,^^42^^,^^44^^,^^47^^,^^71^ that investigated the effects of online training on mental health had significant positive effects on the primary outcome. Significant positive effects were also observed in 3 of the 6 studies (50%) that included smartphone application interventions.^35^^,^^37^^,^^43^

All 5 studies (100%) targeting healthy lifestyle^11^^,^^30^, ^31^, ^32^, ^33^ reported significant results. The interventions used were telecare,^31^^,^^32^ smartphone applications,^11^ online interventions,^33^ and blended interventions.^30^ Regarding cardiovascular health, significant results were observed in all 5 studies (100%).^18^^,^^21^, ^22^, ^23^, ^24^, ^25^ The interventions used smartphone applications alone,^22^ in combination with online training,^25^ or online trainings alone,^21^^,^^24^ and 1 was a blended intervention.^23^ For diabetes, significant results were obtained in 3 of the 4 (75%) interventions that used smartphone applications in combination with wearables, e-mails, messaging, and blended interventions.^27^, ^28^, ^29^

Using the Effective Public Health Practice Project framework (Table 5), the overall quality of the studies (including the evaluation of risk of bias) was rated as high in 7 cases,^19^^,^^21^^,^^30^^,^^50^^,^^51^^,^^61^^,^^67^ moderate in 17 cases,^18^^,^^20^^,^^24^^,^^28^^,^^32^^,^^36^^,^^37^^,^^41^^,^^42^^,^^47^^,^^49^^,^^53^^,^^58^^,^^62^^,^^63^^,^^68^^,^^70^ and weak in 28 cases.^11^^,^^22^^,^^23^^,^^26^^,^^27^^,^^29^^,^^31^^,^^33^, ^34^, ^35^^,^^38^, ^39^, ^40^^,^^43^, ^44^, ^45^, ^46^^,^^48^^,^^54^, ^55^, ^56^, ^57^^,^^59^^,^^60^^,^^64^, ^65^, ^66^^,^^69^ and not applicable in 2.^25^^,^^52^ Potential selection bias was the most commonly identified bias and was frequently acknowledged by the researchers themselves. The generalizability of the results was also a frequently highlighted issue because the study populations were often quite small or highly specific. None of the studies were excluded from the analysis on the basis of the risk of bias. Supplemental Appendix 4 provides further details on the quality of the studies.

**Table 5:** Evaluation of Bias According to Effective Public Health Practice Framework

	Strong	Moderate	Weak	NA
Sedentary behavior	3^50^^,^^51^^,^^61^	4^53^^,^^58^^,^^62^^,^^63^	9^54^, ^55^, ^56^, ^57^^,^^59^^,^^60^^,^^64^, ^65^, ^66^	1^52^
Mental health	0	5^36^^,^^37^^,^^41^^,^^42^^,^^47^	9^34^^,^^35^^,^^38^, ^39^, ^40^^,^^43^, ^44^, ^45^, ^46^	
Mental health and sedentary behavior	1^67^	1^68^	0	
Cardiovascular disease	1^21^	1^24^	2^22^^,^^23^	1^25^
Diabetes	0	1^28^	3^26^^,^^27^^,^^29^	
Healthy behavior	1^30^	1^32^	3^11^^,^^31^^,^^33^	
Access to care	1^19^	1^18^	—	
Process	0	1^49^	1^48^	
Complaints of the arm neck and shoulder	0	1^20^	0	
Sickness absence	0	0	1^69^	
Work ability	0	1^70^	—	

NA, not applicable.

## Discussion

In this systematic review, the aim of which was to assess the existing body of evidence on the impact of digital interventions in occupational health care, we observed that digital intervention research in occupational health care focuses on mental health and sedentary behavior. It appears that online training or interventions may have significant effects, and overall, significant effects were more prominent in the studies of healthy lifestyle, cardiovascular diseases, and mental health. We also concluded that heterogeneity is a significant issue in outcome measures and that it affects whether results can be pooled and more detailed conclusions drawn.

The identified focus of digital interventions targeting mental health and sedentary behavior in occupational health care is in line with that in the existing literature.^3^^,^^5^ It is also in line with the current trends and needs of workplace wellbeing, such as focusing on preventive measures, because sedentary behavior poses the risk of an array of chronic diseases and mental health problems, with the added burden to mental wellbeing brought by global digital developments that can be seen in workplaces today.^3^^,^^6^

According to this review, computer applications and online training often had a positive impact on selected occupational health care outcomes. This was the case in all 5 studies of computer applications and in 8 of 11 studies of online training or interventions. Of the studies primarily focusing on mental health, 11 of 14 studies reported a significant positive effect, whereas of the 16 studies examining sedentary behavior, only 8 reported a significant positive effect, 1 study reported no significance in this matter. Previous systematic reviews of digital intervention studies of both mental health and sedentary behavior have also observed similar small effects.^2^ Digital interventions have been found to have positive effects on mental health; these effects being stronger among workers with initially high stress levels.^72^ Although fewer of the studies in this review focused on healthy lifestyle and cardiovascular health than those on mental health, 5 of each, both health targets show promise, and all 5 healthy lifestyle studies found a significant primary outcome measure, as did the 5 cardiovascular health studies. However, it would be fruitful to further investigate this promising trend in focused reviews using the same metrics because this would enable the comparison of results and provide reliable information on the impact of digital interventions on mental health and other interesting occupational health care focuses.

It was also interesting to note that of the 54 included studies, only 2 had patient experience or engagement as their primary outcome, and 20 of the included studies did not take patient engagement, satisfaction, or experience into consideration at all. As encouragement of patient engagement is recommended, especially in digital interventions, we had hoped that this ratio would have been even higher.^2^ This result should be interpreted with caution because we did not include a specific search term for patient engagement. However, we did not restrict the target of the digital interventions or the chosen outcome for this review.

We were unable to pool the results owing to the wide variations in the selection of health targets and subsequent choice of outcome measures. Previous systematic reviews have also identified the heterogeneity of studies, especially in terms outcome measurement, as an issue. For instance, Howarth et al^2^ found moderate evidence of purely digital interventions, with effects ranging from sleep and mental health to sedentary behavior, but mentioned lack of consensus on outcome measure as hampering stronger evidence.

Regarding the quality of the included studies, a common issue was selection bias, either in the form of voluntary participants who were perhaps more susceptible to changing their habits or behaviors or in obtaining enough participants from the initially chosen population. Additionally, many studies saw attrition and retention rates as issues; these were often influenced by various factors such as lengthy questionnaires or technical issues with the digital intervention components. This can partly be explained by the fact that a portion of the included studies were pilot studies, and thus, the researchers involved were often also the developers of the intervention, seeking a deeper understanding of its effects. Furthermore, their restrictions on individuals with access to digital tools in their field of work or, further still, to smartphones, create inequalities and make the generalizability of the results challenging. Some studies even required a specific type of smartphone for participation in the study,^51^ or even more interestingly, participation in the intervention group,^64^ thereby causing a clear risk of bias. Finally, regarding publication bias, there was a nonsignificant effect regarding the primary outcome in 19 of the 54 studies, which might signal that the risk of publication bias is acceptable. However, closer examination of the significance of all included measures revealed that the ratio changed drastically to nonsignificant effects in only 3 of the 54 studies.

Despite over 2 decades of research and use of digital interventions in occupational health care, there are few signs that developers or researchers take into account existing evidence when developing their projects and reaching consensus on outcome measures as recommended.^9^ To combine results and offer more specific, quantifiable recommendations to health care professionals and providers, occupational health care researchers should really strive for consensus on outcome measures.

Given that mental health, especially that of younger adults, is now a major global concern, all tools that can potentially help reduce the societal disease burdens related to mental health are extremely important.^73^ It is also noteworthy that all studies discussed in this study span the past decade and that not all their participants had as much experience with digital tools in general as the digitally native generations that have increasingly entered the workforce. The potential of these younger generations and its possible implications is something researchers should explore.

### Strengths and Limitations of the Research

We recognize several strengths in our study. One is its broad scope, encompassing both the digital interventions used in the included studies and the health targets addressed. Another important strength was our goal to reflect real-world settings, leading us to include various study types, as long as they consisted of original research. In practice, digital interventions are typically part of larger intervention groups targeting health improvement and are not generally implemented in isolation, as is the case in RCTs. However, because they minimize any confusion caused by other concurrent factors affecting the workers in addition to the interventions, RCTs still play an important role in research.

Our study has certain limitations that should be considered when evaluating the significance of the results. For instance, its inclusive approach may have led to variation in the overall quality of the included studies, at least when assessed using the quality assessment tools available. This variation can be viewed as a limitation of the systematic review. Another limitation is the heterogeneity of the studies, which, as mentioned earlier, prevented us from pooling the results for meta-analysis. Consequently, the outcomes were categorized as significant or not significant rather than presented with actual means with CIs, odds ratios, or similar metrics, leading to a more simplified interpretation of the findings.

In the future, it would be essential for occupational health care researchers to reach consensus on key outcome measures. This would ensure comparable results, thereby providing reliable information for health care professionals. Occupational health care should pay special attention to the possibilities in mental health. Another aspect to consider is whether the focus should be solely on improving difficult outcomes or whether it would be more beneficial to invest in empowering patients and enhancing patient satisfaction, as well as health care professionals’ satisfaction and the usability of the intervention.

## Conclusion

The potential positive impact of digital interventions on mental health, especially online training, should be leveraged by health care professionals and providers in the future. Their potential to support the management of cardiovascular disease and diabetes, and to promote a healthy lifestyle is also noteworthy. Online training is well suited to addressing mental health issues such as burnout and depression in workplace settings, and although they require an initial investment, such interventions are subsequently quite scalable in office environments for instance, where they can have a substantial impact. This offers an excellent opportunity to address one of the key targets of combating the global mental health problem.

## Potential Competing Interests

Dr Matintupa received grants from Finska Läkaresällskapet for her doctoral studies, which was used for the work on this manuscript and received grants from Waldemar von Frenckells stiftelse and Svenska Österbottniska Samfundet. The other authors declare no conflicts of interest.
