# Supplementary material for: Impact of Digital Interventions in Occupational Health Care: A Systematic Review
Source: Mayo Clin Proc Digit Health. 2025 Mar 18;3(2):100216. doi: 10.1016/j.mcpdig.2025.100216 (PMC12190893; doi:10.1016/j.mcpdig.2025.100216)
Supplement: Supplemental Material [file mmc1.pdf]

Impact of digital interventions in occupational healthcare - a systematic review

Appendix 1 List of excluded studies at the full text stage

Authors	Title	Reason for exclusion
Allen et al (2017) [1]	Development of a Diabetes Mellitus Knowledge Resource for Clinical Decision Support Assisting Primary Care Physicians With Work-Related Issues.	Not evaluation of impact of digital intervention
Baker & Jacobs (2014)[2]	The feasibility and accuracy of using a remote method to assess computer workstations.	Not evaluation of impact of digital intervention
Bonn et al (2019) [3]	App-technology to improve lifestyle behaviours among working adults - The Health Integrator study, a randomized controlled trial	Not evaluation of impact of digital intervention
Bos et al (2015) [4]	Evaluating the (cost-)effectiveness of guided and unguided Internet-based self-help for problematic alcohol use in employees--a three arm randomized controlled trial.	Not evaluation of impact of digital intervention
Carter et al (2020) [5]	Using an e-health intervention to reduce prolonged sitting in UK office workers: A randomised acceptability and feasibility study	Not evaluation of impact of digital intervention
Cher et al (2019) [6]	Utility of a Telephone Triage Hotline in Response to the COVID-19 Pandemic: Longitudinal Observational Study.	Other : no evaluation on the impact on patients
De Cocker et al (2018) [7]	From evidence-based research to practice-based evidence: Disseminating a web-based computer-tailored workplace sitting intervention through a health promotion organisation	Not evaluation of impact of digital intervention
De Cocker et al (2015) [8]	Theory-driven, web-based, computer-tailored advice to reduce and interrupt sitting at work: Development, feasibility and acceptability testing among employees	Not evaluation of impact of digital intervention
De Cocker et al (2017) [9]	What are the working mechanisms of a web-based workplace sitting intervention targeting psychosocial factors and action planning?	Not evaluation of impact of digital intervention
Dillane & Balanay (2020) [10]	Comparison between OSHA-NIOSH Heat Safety Tool app and WBGT monitor to assess heat stress risk in agriculture.	Not evaluation of impact of digital intervention
Edgerton et al (2017) [11]	A Pilot Study Investigating Employee Utilization of Corporate Telehealth Services	Not evaluation of impact of digital intervention
Geraedts et al (2013) [12]	Web-based guided self-help for employees with depressive symptoms (Happy@Work): Design of a randomized controlled trial	Not evaluation of impact of digital intervention
Girerd et al (2019) [13]	Development of the Depist'HTA R score to predict a rise in blood pressure during a self-testing session: PREDIC-HTA bakery survey.	Not evaluation of impact of digital intervention
Gleason (2021) [14]	Remote Monitoring of a Work-From-Home Employee to Identify Stress: A Case Report	Not correct study type
Goetzel et al (2017) [15]	Prevalence of Metabolic Syndrome in an Employed Population as Determined by Analysis of Three Data Sources.	No digital intervention (according to study definition)
Green et al (2020) [16]	Rapid Deployment of Chiropractic Telehealth at 2 Worksite Health Centers in Response to the COVID-19 Pandemic: Observations from the Field.	Not evaluation of impact of digital intervention
Greenfield et al (2016) [17]	Truck drivers' perceptions on wearable devices and health promotion: a qualitative study.	Not evaluation of impact of digital intervention
Henny et al (2013) [18]	The Business case for telemedicine	Not correct study type
Hutting et al (2015) [19]	Development of a self-management program for employees with complaints of the arm, neck, and/or shoulder: An intervention mapping approach	Not evaluation of impact of digital intervention
Jesuthasan et al (2022) [20]	The Impact of Personalized Human Support on Engagement With Behavioral Intervention Technologies for Employee Mental Health: An Exploratory Retrospective Study.	Not evaluation of impact of digital intervention
Jimenez & Bregenzer (2018) [21]	Integration of eHealth Tools in the Process of Workplace Health Promotion: Proposal for Design and Implementation.	Not evaluation of impact of digital intervention
Kitchingman et al (2018) [22]	The impact of their role on telephone crisis support workers' psychological wellbeing and functioning: Quantitative findings from a mixed methods investigation	Not evaluation of impact of digital intervention
Langlieb et al (2021) [23]	EAP 2.0 reimagining the role of the employee assistance program in the new workplace	Not evaluation of impact of digital intervention
Leiter et al (2018) [24]	The relationship of safety with burnout for mobile health employees	No digital intervention (according to study definition)
Maciel et al (2019) [25]	Development of an e-health education program at the workplace using formative research – Technologies for improving quality of life	Not evaluation of impact of digital intervention
Marovich et al (2020) [26]	Opportunities at the intersection of work and health: Developing the occupational data for health information model.	Not evaluation of impact of digital intervention
Marques & Pitarma (2019) [27]	MHealth: Indoor environmental quality measuring system for enhanced health and well-being based on internet of things	Not evaluation of impact of digital intervention
Milligan-Saville et al (2017) [28]	Workplace mental health training for managers and its effect on sick leave in employees: a cluster randomised controlled trial	No digital intervention (according to study definition)
Naranjo-Saucedo et al (2023) [29]	Mobile Health Requirements for the Occupational Health Assessment of Health Care Professionals: Delphi Study.	Not evaluation of impact of digital intervention
Nelson et al (2021) [30]	Pilot Feasibility Study of Incorporating Whole Person Care Health Coaching Into an Employee Wellness Program	Other : Not occupational healthcare

Neyens & Childers (2017) [31]	Determining Barriers and Facilitators Associated with Willingness to Use a Personal Health Information Management System to Support Worksite Wellness Programs	No digital intervention (according to study definition)
Park et al (2020) [32]	Development and Operation of a Video Teleconsultation System Using Integrated Medical Equipment Gateway: a National Project for Workers in Underserved Areas.	No digital intervention (according to study definition)
Persechino et al (2013) [33]	Work-related stress risk assessment in Italy: a methodological proposal adapted to regulatory guidelines.	Not evaluation of impact of digital intervention
Reyes et al (2016) [34]	An Evaluation Tool for Agricultural Health and Safety Mobile Applications.	Not evaluation of impact of digital intervention
Rose et al (2021) [35]	Patient Experience in Virtual Visits Hinges on Technology and the Patient-Clinician Relationship: A Large Survey Study With Open-ended Questions.	Other : Not occupational healthcare
Siemiatycki (2018) [36]	Availability of a New Job-Exposure Matrix (CANJEM) for Epidemiologic and Occupational Medicine Purposes.	No digital intervention (according to study definition)
Stephenson et al (2021) [37]	The “Worktivity” mHealth intervention to reduce sedentary behaviour in the workplace: a feasibility cluster randomised controlled pilot study	Not evaluation of impact of digital intervention
Suman et al (2018) [38]	Effectiveness of a multifaceted implementation strategy compared to usual care on low back pain guideline adherence among general practitioners	Other - not occupational healthcare
Syrjala et al (2021) [39]	Reducing occupational sitting time in adults with type 2 diabetes: Qualitative experiences of an office-adapted mHealth intervention.	Not evaluation of impact of digital intervention
Szalewska et al (2015) [40]	The impact of professional status on the effects of and adherence to the outpatient followed by home-based telemonitored cardiac rehabilitation in patients referred by a social insurance institution.	Not evaluation of impact of digital intervention
Tajvar et al (2022) [41]	Developing a Decision Aid Tool for selecting pen-paper observational ergonomics techniques: a quasi-experimental study.	Not evaluation of impact of digital intervention
Tchir & Szafron (2020) [42]	Occupational Health Needs and Predicted Well-Being in Office Workers Undergoing Web-Based Health Promotion Training: Cross-Sectional Study.	Not evaluation of impact of digital intervention
Vahteristo & Jylha (2020) [43]	Effects of User Participation in the Development of Health Information Systems on Their Evaluation Within Occupational Health Services.	Not evaluation of impact of digital intervention
Volker et al (2013) [44]	Blended E-health module on return to work embedded in collaborative occupational health care for common mental disorders: design of a cluster randomized controlled trial.	Not evaluation of impact of digital intervention
Volker et al (2017) [45]	Process Evaluation of a Blended Web-Based Intervention on Return to Work for Sick-Listed Employees with Common Mental Health Problems in the Occupational Health Setting	No digital intervention (according to study definition)
Wattenberg et al (2015) [46]	Assessment of the Acute and Chronic Health Hazards of Hydraulic Fracturing Fluids.	No digital intervention (according to study definition)
Weichelt et al (2019) [47]	Farm Owners and Workers as Key Informants in User-Centered Occupational Health Prototype Development: A Stakeholder-Engaged Project.	Not evaluation of impact of digital intervention
Werkmeister et al (2023) [48]	Experiences of the COVID-19 Lockdown and Telehealth in Aotearoa New Zealand: Lessons and Insights from Mental Health Clinicians	Not evaluation of impact of digital intervention
White et al (2013) [49]	Comparison of cardiovascular risk calculation tools in pharmacy practice.	Not evaluation of impact of digital intervention
Yang et al (2018) [50]	Towards Smart Work Clothing for Automatic Risk Assessment of Physical Workload	Not evaluation of impact of digital intervention
Ye et al (2021) [51]	Development of a chatbot program for follow-up management of workers’ general health examinations in korea: A pilot study	Not evaluation of impact of digital intervention
Zamora-Ilarionov & Rodriguez (2020) [52]	The use of eHealth to design a regional health promotion program in the workplace: Institute of Costa Rican Electricity case series	Not evaluation of impact of digital intervention

Appendix 2 SERCH STRATEGY

Search string for SCOPUS 5.6.2023:

(TITLE-ABS-KEY ("digital* health*" OR "m-health*" OR mhealth* OR telemedicine* OR "e-health*" OR ehealth* OR "health-IT*" OR "virtual* health*")) AND (TITLE-ABS-KEY ("occupational* healthcare*" OR "occupational* health*" OR "employee* health*")) AND (TITLE-ABS-KEY (impact* OR assess* OR value* OR efficiency* OR effect*)) AND (LIMIT-TO (PUBYEAR , 2023) OR LIMIT-TO (PUBYEAR , 2022) OR LIMIT-TO (PUBYEAR , 2021) OR LIMIT-TO (PUBYEAR , 2020) OR LIMIT-TO (PUBYEAR , 2019) OR LIMIT-TO (PUBYEAR , 2018) OR LIMIT-TO (PUBYEAR , 2017) OR LIMIT-TO (PUBYEAR , 2016) OR LIMIT-TO (PUBYEAR , 2015) OR LIMIT-TO (PUBYEAR , 2014) OR LIMIT-TO (PUBYEAR , 2013))

382 finds originally and after limiting to 10 years 287 results

Search strategy for Ovid MEDLINE 5.6.2023:

1	mhealth.mp. or exp Telemedicine/	50143
2	mobile health.mp.	12147
3	telemedicine.mp.	48292
4	e-health.mp.	4144
5	ehealth.mp.	6940
6	exp Medical Informatics/ or health	502398
7	virtual health.mp.	951
8	digital health.mp.	7436
9	m-health.mp. or exp Mobile Applic	12051
10	1 or 2 or 3 or 4 or 5 or 6 or 7 or 8 c	578775
11	exp Occupational Health/ or exp C	46403
12	occupational health.mp.	60581
13	employee health.mp.	1580
14	11 or 12 or 13	61425
15	impact.mp.	1344788
16	assessment.mp.	1685900
17	value.mp.	1391303
18	exp Efficiency, Organizational/ or e	611214
19	15 or 16 or 17 or 18	4544291
20	10 and 14 and 19	441

441 results originally and after limiting to last 10 years 234 results

Appendix 3 Excluded studies at data extraction

Author and publication	Title	Study Design	Reason
Guo et al (2016) [53]	A Big-Data-based platform of workers' behavior: Observations from the field.	Case study	The design, data collection and studies form this study was completely different from the other studies and as such not much relevant data could be extracted and decision was made to leave the study out.
Manghisi et al (2020) [54]	A body tracking-based low-cost solution for monitoring workers' hygiene best practices during pandemics.	Validation study	As this was a validation study of a body tracking solution focus and the data was not complimentary to our data extraction model and the decision was made to leave the study out.
Martin et al (2020) [55]	Protecting the Mental Health of Small-to-Medium Enterprise Owners: A Randomized Control Trial Evaluating a Self-Administered Versus Telephone Supported Intervention	RCT	Only telephone discussions and DVD and as such was not relevant digital focus
Morris et al (2021) [56]	Rise and recharge: Exploring employee perceptions of and contextual factors influencing an individual-level e-health smartphone intervention to reduce office workers' sedentary time at work	Feasibility study	Another report from the same Rise and recharge project was included in the final analysis, however this report was a feasibility study and as such the focus on impact that is the focus for the review was not filled and this report was left out of the final analysis.
Peter et al (2019) [57]	Effectiveness of an Online CBT-I Intervention and a Face-to-Face Treatment for Shift Work Sleep Disorder: A Comparison of Sleep Diary Data.	Comparative study	This study compared sleep diaries between online and outpatient samples and further analysis of the online sample was also conducted. The synthesis of data and overall approach did not match the studies included in the review and as such the study was left out from the final analysis.

Abbreviations:

RCT Randomized controlled trial

Appendix 4 SERCH STRATEGY

Authors	Quality of study (evaluation of bias)	Comments
Atkins et al (2020)[58]	Weak	Change to the national reporting of sickness absence during the period might affect the results<br>Physician turnaround during the period might mean that information about the process did not meet all and as such cause bias<br>Pragmatic design, potential crossover of physicians from intervention to control.<br>During the study period the occupational healthcare provider acquired another healthcare provider - this was taken into consideration in final analysis.
Balk-Møller et al (2017)[59]	Weak	Power calculations done, however drop out a bit more than expected which might have affected the statistical power, Analysis of differences between populations done (clinical, SES)<br>Large sample from different regions and varied demographic factors so generalizability OK. Also as many as possible not only motivated workers, which a lot of other studies have. nonuser group in the intervention group as participants not all familiar or comfortable with digital tools.<br>Non-user group in the intervention group (had not used mobile phones before, ow ses)<br>The randomization, retention rate low
Boerema et al (2019)[60]	Weak	Small sample - from the university<br>Some of the participants were familiar with physical activity research and were, of course, aware of the fact that they were being<br>monitored as part of our study (as we did not conduct a classic randomised controlled trial which would have<br>accounted for this effect).<br>Short time - no sustained effect<br>Time of year<br>No control
Bolier et al (2014)[61]	Weak	Uptake and compliance was very low which affects the reliability of the results.<br>Participants required for adequate statistical power was not fully achieved.<br>High dropout overall and also differences between groups that might lead ti selection bias.
Bort-Roig, et al (2020)[62]	Weak	Selection of participants - only if they were interested and had access to certain smartphones<br>Active control - both groups<br>had access to a mobile phone application (the IG had access to the full W@W-App while the AC-G<br>had access to a partial W@W-App with only the self-monitoring features opened) a<br>complete control would have been good<br>high rate of<br>High dropout - battery issues<br>Not enough description of the populations and were the groups comparable?<br>They measured at lot pre and post which were significant but the difference between groups was smaller
Carr & Kevitt (2023)[63]	Weak	Limitations included discrepancies with calculated travel times, both regarding arrival and taken into consideration only driving not public transport<br>Covid might affect as the timing was when in-person visits were only starting<br>No validated questionnaire for user satisfaction
Chen et al (2019)[64]	Strong	Self-reported outcomes (recall bias and reporting bias)<br>Localized so generalizability cannot be guaranteed
Comtois et al (2022)[65]	Strong	Short - 4 weeks, participants for this study was part of a larger study- needed to own a smartphone so this creates issues with generalizability,<br>Pragmatic randomized trial -3 apps and an attention control app . It was considered unethical to have a waitlist or placebo control.<br>Demographic factors taken into consideration in baseline measures. Blindness not fully achieved and some participants reported use of the other apps too.<br>Self-reported use as they did not partner with the app.-manufacturers.<br>Study conducted in 2020 during COVID-19 epidemic
Cooley et al (2014)[66]	NA	Quite small sample, only 15 for qualitative interviews, so no real comparison and not RCT
Costa et al (2022)[67]	Moderate	The study was funded by SWORD Health that created the intervention<br>No control, loads of measures, self-reported outcomes<br>Several demographic characteristics were different between clusters,
De Kock et al (2022)[68]	Moderate	Attrition rate might affect the results (participants not as satisfied or not seeing results might have dropped out more)<br>Small sample and single site, generalizability might be affected<br>Cross-contaminations effects as app free to download and participants not restricted to not use other means
Ebert et al (2018)[69]	Moderate	Followed guidelines from the ISPOR RCT-CEA Task Force Report

		and the recommendations of the Consolidated Health Economic Evaluation Reporting Standard (CHEERS)<br>Even though participants were not excluded it they didn't belong to a large health insurance company the recruitment was largely targeted via this company<br>Limitations of our study include the fact that our largescale trial was still underpowered for economic analyses<br>RCT was not targeted for this as this was only part of it<br>Pop not applicable as only persons with server stress included and self-selection might cause bias<br>Authors are stakeholders in the instituted that promotes use of solutions that were examined
Gayed et al (2019)[70]	Strong	Measures were decided on priori to the trial (following trail protocol),<br>self-reported questionnaires<br>retention rate not the best<br>Choice of primary outcome interesting as it is indirect and no direct impact could be seen - perhaps to short<br>Manager linkage to worker was not direct, and this is a definite risk for bias<br>Researchers created intervention
Gilson et al (2017)[71]	Weak	Target population based on sample calculations not reached so detection of significant outcomes affected and also generalizability affected.<br>Incentives involved.<br>Lack of control group
Gwain et al (2022)[72]	Moderate	Small sample and short time, self-reported outcomes<br>No control only pre and post intervention survey
Haile et al (2020)[73]	Weak	Small sample size so generalizability not great<br>ActivePal given to participants might have had additional stimulation to move
Huang et al (2023)[74]	Weak	Baseline analysis showed a significant difference in age.<br>Cluster randomization<br>Self-reported exercise.<br>Quasi experimental design might cause differences between groups.<br>Small, local sample so generalizability not high
Hutting et al (2015)[19]	Strong	Low utilization of the eHealth might impact and the non sig results (17 % did not use for the first 3 months and 66 %did not use after the first 3 months) And none used it daily.<br>Blindness to allocation to group could not be achieved. Participants were largely high educated and in healthcare which might affect the generalizability and the results. Drop out might have led to selection bias- a lot of persons in CAU dropped out as it was considered very time consuming to fill out the forms.
Johnson et al (2021)[75]	Weak	No control group, sometimes self-reported outcomes, generalizability might be affected as there was only one employment group
Judice et al (2015)[76]	Weak	crossover - those that had the intervention first hard to believe it did not impact their activity during the control period - no wash-out<br>No separate control group, small sample size so generalizability might be affected
Jukic et al (2020)[77]	Weak	Small size so generalizability affected. Also, short time period so the permanency of results cannot be determined<br>No control group<br>Large array of outcome measures<br>Participants volunteered so possible selection bias of more motivated individuals<br>The company that developed the app used in the study also helped fund the study
Kempf et al (2019)[78]	Moderate	Difference in drop out in the different arms, this might have led to selection bias as only highly motivated individuals remained in the intervention arm.<br>Due to legal issues demographic characteristics were not allowed to be collected which might have affected the results.<br>Missing data considered in analysis but might have affected.
Kouwenhoven-Pasmooij et al (2017)[79]	Strong	Researchers created the intervention themselves<br>No control group<br>No pre-intervention measures for the physical activity (only self-reported)<br>Lack of power test<br>Logarithmic regression used<br>Targeted population (motivated to change behaviour)<br>Programme free for 24 first participants and then 450 euros for the next 28<br>Study not adequately equipped to measure weightloss (to short)<br>CONSORT followed
Kouwenhoven-Pasmooij et al (2018)[80]	Weak	response rate high compared to other studies<br>lack of control both arms different forms of the intervention, cluster randomization - different sizes clusters, baseline evaluation showed differences in clusters - adjustment for statistical analysis might affect the result<br>self-rated health first outcome
Lau & Faulkner (2019)[81]	Weak	No randomization, no control<br>No consideration of potential confounders regarding the physical layout of the workplace<br>Focus only on core components<br>Use of Garmin vivo fit might affect the results - extra motivation
Lavaysse et al (2022)[82]	Weak	Authors are associated with Onedrop<br>Self-reported outcomes<br>Older population so not widely generalizable

		Exclusion of some participants for analysis, with low interaction with the intervention might cause bias
Lee et al (2019)[83]	Moderate	Small sample and short time so generalizability and permanence of results might be affected<br>No randomization.<br>Possible effect if Fitbit itself
Lennefer et al (2020)[84]	Weak	As no control group intervention effects were influenced by contextual factors (e.g., time of the year when the study started To reduce this confounding<br>influence, we conducted the intervention in two randomized<br>groups that participated consecutively in the intervention<br>activities, making the possible influence of the time of year<br>less likely.<br>activity data collected by the activity tracker to operationalize physical activity, however it was still self-reported due to regulations<br>Sample size hard to estimate as no similar studies<br>the sample was recruited from only one company in Germany - applicability problems<br>Because we do not have any data on how employees<br>used the features of the activity tracker, future studies<br>should further investigate which features are effective in<br>improving impaired well-being among employees.
Lokmann et al (2017)[85]	Weak	High loss to follow up (selection bias), missing data. Taken to consideration in analysis but might still affect.<br>Self-reported outcomes<br>Study powered to detect differences in sickness absence not economic factors and as such the CI are large. Cost at baseline differed sig between groups
MacDonald et al (2020)[86]	Weak	Dropout rate high - technical issues affected one company which was then excluded in analysis<br>Subjective measure of sed behaviour<br>No control<br>Focus groups for only part of the participant (16)<br>Focus on reach and adoption not only impact<br>Apparently smaller companies were more likely to take part and larger companies even though contacted choose not to take part which might affect the applicability
Mainsbridge et al (2014)[87]	Moderate	Outcomes regarding exercise based on self report, possible selection bias as it might be that people that already have the motivation to live healthier joined<br>Retention rate not ultimate
Mainsbridge et al (2018)[88]	Weak	Generalizability might be affected as a large number of participants were female.<br>Small sample so detection of all outcomes might have been affected also to conduct sub analysis to minimize the effect of demographic factor which could not be done here.<br>Participants not matched on demographic factors.<br>Random calls to participants during the study - asking about the programme and daily routine - might this also have served as a motivator and reminder and as such affected the results.<br>Research team tried to blind participants by not telling them the study hypothesis. Not full blindness as the intervention was seen but Hawthorne effect was mitigated by telling control group participants that they would revive the intervention after a time period.
Maylor et al (2018)[89]	Strong	Cluster randomization<br>keeping apart cluster and participants when discussing progression of study, sensitivity analysis conducted (only included people with a certain number of active PAL (tracker) wear time
Meyer et al (2018)[90]	Weak	No control group, Low response rate to the surveys, especially the final one. Tried to take into consideration in analysis but might still affect<br>IPV to decrease estimation bias
Michelsen & Kjellgren (2022)[91]	Weak	As this was a combined study from different countries it was no preregistered at clinicaltrials.gov as it was approved by a Swedish regional ethics committee-<br>Quite small sample so generalizability might be affected.
Morris et al (2020)[92]	Weak	Quasi randomization (eg. Iphone user vs other smartphones)<br>Contamination between arms possible and heterogenicity between groups<br>not powered to detect changes in behavioural or cardiometabolic outcomes.
Muniswamy et al (2022)[93]	Weak	Poor engagement with the intervention might impact the results, Hawthorne effect - access to other sources of information
Nagata et al (2022)[94]	Moderate	Rigid baseline characteristics palette<br>First, there were a large volume of missing data in the questionnaires and<br>biochemical tests. This might cause bias<br>Participants n this study were likely to have a high level of awareness of<br>behaviour change, this affects the interpretation of results
Notenbomer et al (2018)[95]	Moderate	Adherence to the tool was low (low motivation) and a large amount of participants did not even read all the instructions.<br>the control group was made aware of frequent SA and the risk of long-term SA by the invitational study leaflet and the Web-based questionnaire so that they might have gotten more information and as such caused a smaller effect. - Take more actions
Nundy et al (2014)[96]	Weak	Qasi-experimental design<br>Demographic analysis and baseline characteristics analysed<br>Selection bias: control group were the ones that did not have mobile phones or did not

		answer recruitment emails
Park et al (2022)[97]	Moderate	No randomization and no matching of subject on demographic characteristics as part of national project.<br>Short term so permanence of effects not sure<br>For generalizability larger nationwide sample needed<br>Set during covid-19 so this might also affect selection
Pedersen et al (2014)[98]	Moderate	Pilot - so sample size small<br>To prevent any type of Hawthorne effect, participants in the control group were told that at the conclusion of the first 13-week intervention period they too would receive the e-health software for<br>a second 13-week intervention period<br>Self-reported outcomes (working hours)<br>Most studies on the subject use other questionnaire international Physical Activity Questionnaire but researchers created their own since it does not differentiate between different sources of energy expentiryre<br>Short time period so sustainability
Ryu et al (2021)[99]	Moderate	Power calculations done,<br>Demographics compared between groups satisfactory, not generalizable (too small) too short to see any significant changes
Röhling et al (2020)[100]	Moderate	Invitation to take part could lead to selection bias as only motivated individuals join.<br>the imputation<br>Reporting bias might be due to LOCF approach for missing values -underestimation<br>Small sample (sample size calculations conducted) might lead to decreased detection and lack of generalizability
Sasaki et al (2021)[101]	Strong	Generalizability only to nursing and people with access to smartphone<br>Adequate completion rate<br>Self-reported outcomes<br>Contamination of information to the control group possible<br>Group chat (informal) with the persons in the intervention groups, researchers and head nurses might affect work engagement<br>Frustration with reminders might also affect the results
Simons et al (2017)[102]	Weak	no flow of participant chart (although retention not 100), no demographics of participants (available from authors)<br>Generalizability only for people motivated to make a change<br>In waiting control joined intervention after 6 weeks causing a demotivation effect<br>Designed to test after 6 weeks not later so all other not as reliable
Thogersen-Ntoumani et al (2020)[103]	Moderate	Pilot study and not powered to detect significant effects for all outcomes<br>Fitbit trackers given at baseline- might cause bias<br>Demographic factors taken into consideration, interpretation somewhat difficult<br>A variety of measures, and a lot of sub analysis considering the sample size<br>Dropout rate low
Umanodan et al (2014)[104]	Moderate	Cluster randomization<br>A lot of measures and sub analysis<br>Population from research and development (mostly) so generalizability might be affected<br>Self-reported measures<br>Baseline measures conducted; significant difference regards to work engagement in favour of the intervention group<br>Non completers had higher scores regarding distress in its intervention group which might lead to bias<br>No knowledge about how long it takes for the intervention effect to appear
Van Schaaijk et al (2019)[105]	Weak	Small sample (trouble with recruiting participants)<br>Use of intervention very low which might affect the reliability of the results<br>Lack of information on reasons for dropout might lead to selection bias<br>Change of analysis when no effects between groups to analyse as a group as a whole.
Volker et al (2015)[106]	Moderate	Low adherence - especially amongst the occupational physicians that were to take part in the study but also amongst the patients.<br>Power calculations done (over 200 needed). Demographic factors taken into consideration.<br>Randomization only pseudo for one of the sites - participants that completed the first questionnaire.<br>Bias a loss to follow up larger in the intervention group than CAU
Widmer et al (2014)[107]	Weak	Financial benefits (preferable insurance plan) might affect the results<br>Use of survey data and not direct data<br>No randomization and no control group<br>No data on the excluded/drop outs which might lead to selection bias<br>Authors are employed by the ones who own the intervention
Widmer et al (2016)[108]	Moderate	Not randomized, lack of control group.<br>Large group and demographic factors analysed.<br>Changes in medications not measured and as such might affect the results.<br>Utilizing survey data.<br>Some of the authors employed by the company that developed the digital health intervention
Willman (2023)[109]	na	Only clinician view<br>Only one author

Wipfli et al (2019)[110]	Weak	Process evaluation - focus only on the three primary outcomes from the whole programme.<br>Two of the authors have ties to the company that created and might benefit from the intervention

Sources:

1. Allen A, Welch L, Kirkland K, Trout D, Baron S. Development of a Diabetes Mellitus Knowledge Resource for Clinical Decision Support Assisting Primary Care Physicians With Work-Related Issues. *J Occup Environ Med*. 2017;59(11):e236-e239. doi:10.1097/JOM.0000000000001181

2. Baker NA, Jacobs K. The feasibility and accuracy of using a remote method to assess computer workstations. *Hum Factors*. 2014;56(4):784-788.

3. Bonn SE, Löf M, Östenson CG, Trolle Lagerros Y. App-technology to improve lifestyle behaviors among working adults - The Health Integrator study, a randomized controlled trial. *BMC Public Health*. 2019;19(1). doi:10.1186/s12889-019-6595-6

4. Bos L, Lehr D, Berking M, Riper H, Schaub MP, Ebert DD. Evaluating the (cost-)effectiveness of guided and unguided Internet-based self-help for problematic alcohol use in employees--a three arm randomized controlled trial. *BMC Public Health*. 2015;15(100968562):1043. doi:10.1186/s12889-015-2375-0

5. Carter SE, Draijer R, Maxwell JD, et al. Using an e-health intervention to reduce prolonged sitting in UK office workers: A randomised acceptability and feasibility study. *Int J Environ Res Public Health*. 2020;17(23):1-21. doi:10.3390/ijerph17238942

6. Cher BAY, Wilson EA, Pinsky AM, et al. Utility of a Telephone Triage Hotline in Response to the COVID-19 Pandemic: Longitudinal Observational Study. *J Med Internet Res*. 2021;23(11):e28105. doi:10.2196/28105

7. De Cocker K, Cardon G, Bennie JA, Kolbe-Alexander T, De Meester F, Vandelanotte C. From evidence-based research to practice-based evidence: Disseminating a web-based computer-tailored workplace sitting intervention through a health promotion organisation. *Int J Environ Res Public Health*. 2018;15(5). doi:10.3390/ijerph15051049

8. De Cocker K, De Bourdeaudhuij I, Cardon G, Vandelanotte C. Theory-driven, web-based, computer-tailored advice to reduce and interrupt sitting at work: Development, feasibility and acceptability testing among employees. *BMC Public Health*. 2015;15(1). doi:10.1186/s12889-015-2288-y

9. De Cocker K, De Bourdeaudhuij I, Cardon G, Vandelanotte C. What are the working mechanisms of a web-based workplace sitting intervention targeting psychosocial factors and action planning? *BMC Public Health*. 2017;17(1). doi:10.1186/s12889-017-4325-5

10. Dillane D, Balanay JAG. Comparison between OSHA-NIOSH Heat Safety Tool app and WBGT monitor to assess heat stress risk in agriculture. *J Occup Environ Hyg*. 2020;17(4):181-192. doi:10.1080/15459624.2020.1721512

11. Edgerton SS. A Pilot Study Investigating Employee Utilization of Corporate Telehealth Services. *Perspect Health Inf Manag*. 2017;14(Fall). https://www.scopus.com/inward/record.uri?eid=2-s2.0-85049264209&partnerID=40&md5=044a463b2494ff4633870158bfceba0a

12. Geraedts AS, Kleiboer AM, Wiezer NM, van Mechelen W, Cuijpers P. Web-based guided self-help for employees with depressive symptoms (Happy@Work): Design of a randomized controlled trial. *BMC Psychiatry*. 2013;13. doi:10.1186/1471-244X-13-61

13. Girerd X, Boualit R, Hanon O. Development of the Depist'HTA R score to predict a rise in blood pressure during a self-testing session: PREDIC-HTA bakery survey. *Ann Cardiol Angeiol (Paris)*. 2019;68(4):237-240. doi:10.1016/j.ancard.2019.08.001

14. Gleason AM. Remote Monitoring of a Work-From-Home Employee to Identify Stress: A Case Report. *Workplace Health Saf*. 2021;69(9):419-422. doi:10.1177/2165079921997322

15. Goetzel RZ, Kent K, Henke RM, et al. Prevalence of Metabolic Syndrome in an Employed Population as Determined by Analysis of Three Data Sources. *J Occup Environ Med*. 2017;59(2):161-168. doi:10.1097/JOM.0000000000000931

16. Green BN, Pence TV, Kwan L, Rokicki-Parashar J. Rapid Deployment of Chiropractic Telehealth at 2 Worksite Health Centers in Response to the COVID-19 Pandemic: Observations from the Field. *J Manipulative Physiol Ther*. 2020;43(5):404.e1-404.e10. doi:10.1016/j.jmpt.2020.05.008

17. Greenfield R, Busink E, Wong CP, et al. Truck drivers' perceptions on wearable devices and health promotion: a qualitative study. *BMC Public Health*. 2016;16(100968562):677. doi:10.1186/s12889-016-3323-3

18. Henny C, Hartington K, Scott S, Tveiten A, Canals L. The business case for telemedicine. *Int Marit Health*. 2013;64(3):129-135.

19. Hutting N, Detaille SI, Engels JA, Heerkens YF, Staal JB, Nijhuis-van der Sanden MWG. Development of a self-management program for employees with complaints of the arm, neck, and/or shoulder: An intervention mapping approach. *J Multidiscip Healthc*. 2015;8:307-320. doi:10.2147/JMDH.S82809

20. Jesuthasan J, Low M, Ong T. The Impact of Personalized Human Support on Engagement With Behavioral Intervention Technologies for Employee Mental Health: An Exploratory Retrospective Study. *Front Digit Health*. 2022;4(101771889):846375. doi:10.3389/fdgth.2022.846375

21. Jimenez P, Bregenzer A. Integration of eHealth Tools in the Process of Workplace Health Promotion: Proposal for Design and Implementation. *J Med Internet Res*. 2018;20(2):e65. doi:10.2196/jmir.8769

22. Kitchingman TA, Caputi P, Woodward A, Wilson CJ, Wilson I. The impact of their role on telephone crisis support workers' psychological wellbeing and functioning: Quantitative findings from a mixed methods investigation. *PLoS ONE*. 2018;13(12). doi:10.1371/journal.pone.0207645

23. Langlieb AM, Langlieb ME, Xiong W. EAP 2.0: reimagining the role of the employee assistance program in the new workplace. *Int Rev Psychiatry Abingdon Engl*. 2021;33(8):699-710. doi:10.1080/09540261.2021.2013172

24. Leiter MP, Jackson L, Bourgeault I, et al. The relationship of safety with burnout for mobile health employees. *Int J Environ Res Public Health*. 2018;15(7). doi:10.3390/ijerph15071461

25. Maciel RRBT, Chiavegato LD, Marin LS, et al. Development of an e-health education program at the workplace using formative research – Technologies for improving quality of life. *Eval Program Plann*. 2019;73:129-137. doi:10.1016/j.evalprogplan.2018.12.009

26. Marovich S, Luensman GB, Wallace B, Storey E. Opportunities at the intersection of work and health: Developing the occupational data for health information model. *J Am Med Inform Assoc JAMIA*. 2020;27(7):1072-1083. doi:10.1093/jamia/ocaa070

27. Marques G, Pitarma R. MHealth: Indoor environmental quality measuring system for enhanced health and well-being based on internet of things. *J Sens Actuator Netw*. 2019;8(3). doi:10.3390/jsan8030043

28. Milligan-Saville JS, Tan L, Gayed A, et al. Workplace mental health training for managers and its effect on sick leave in employees: a cluster randomised controlled trial. *Lancet Psychiatry*. 2017;4(11):850-858. doi:10.1016/S2215-0366(17)30372-3

29. Naranjo-Saucedo AB, Escobar-Rodriguez GA, Tabernero C, Cuadrado E, Parra-Calderon CL, Arenas A. Mobile Health Requirements for the Occupational Health Assessment of Health Care Professionals: Delphi Study. *JMIR Form Res*. 2023;7(101726394):e40327. doi:10.2196/40327

30. Nelson A, Moses O, Rea B, et al. Pilot Feasibility Study of Incorporating Whole Person Care Health Coaching Into an Employee Wellness Program. *Front Public Health*. 2021;8. doi:10.3389/fpubh.2020.570458

31. Neyens DM, Childers AK. Determining Barriers and Facilitators Associated with Willingness to Use a Personal Health Information Management System to Support Worksite Wellness Programs. *Am J Health Promot*. 2017;31(4):310-317. doi:10.4278/ajhp.140514-QUAN-204

32. Park HS, Kim KI, Soh JY, et al. Development and Operation of a Video Teleconsultation System Using Integrated Medical Equipment Gateway: a National Project for Workers in Underserved Areas. *J Med Syst*. 2020;44(11):194. doi:10.1007/s10916-020-01664-w

33. Persechino B, Valenti A, Ronchetti M, et al. Work-related stress risk assessment in Italy: a methodological proposal adapted to regulatory guidelines. *Saf Health Work*. 2013;4(2):95-99. doi:10.1016/j.shaw.2013.05.002

34. Reyes I, Ellis T, Yoder A, Keifer MC. An Evaluation Tool for Agricultural Health and Safety Mobile Applications. *J Agromedicine*. 2016;21(4):301-309. doi:10.1080/1059924X.2016.1211054

35. Rose S, Hurwitz HM, Mercer MB, et al. Patient Experience in Virtual Visits Hinges on Technology and the Patient-Clinician Relationship: A Large Survey Study With Open-ended Questions. *J Med Internet Res*. 2021;23(6):e18488. doi:10.2196/18488

36. Siemiatycki J, Lavoue J. Availability of a New Job-Exposure Matrix (CANJEM) for Epidemiologic and Occupational Medicine Purposes. *J Occup Environ Med*. 2018;60(7):e324-e328. doi:10.1097/JOM.0000000000001335

37. Stephenson A, Garcia-Constantino M, Murphy MH, McDonough SM, Nugent CD, Mair JL. The "Worktivity" mHealth intervention to reduce sedentary behaviour in the workplace: a feasibility cluster randomised controlled pilot study. *BMC Public Health*. 2021;21(1). doi:10.1186/s12889-021-11473-6

38. Suman A, Schaafsma FG, Van De Ven PM, et al. Effectiveness of a multifaceted implementation strategy compared to usual care on low back pain guideline adherence among general practitioners. *BMC Health Serv Res*. 2018;18(1). doi:10.1186/s12913-018-3166-y

39. Syrjala MB, Fharm E, Dempsey PC, Nordendahl M, Wennberg P. Reducing occupational sitting time in adults with type 2 diabetes: Qualitative experiences of an office-adapted mHealth intervention. *Diabet Med J Br Diabet Assoc*. 2021;38(6):e14514. doi:10.1111/dme.14514

40. Szalewska D, Niedoszytko P, Gierat-Haponiuk K. The impact of professional status on the effects of and adherence to the outpatient followed by home-based telemonitored cardiac rehabilitation in patients referred by a social insurance institution. *Int J Occup Med Environ Health*. 2015;28(4):761-770. doi:10.13075/ijomeh.1896.00494

41. Tajvar A, Daneshmandi H, Seif M, Parsaei H, Choobineh A. Developing a Decision Aid Tool for selecting pen-paper observational ergonomics techniques: a quasi-experimental study. *Med Lav*. 2022;113(5):e2022042. doi:10.23749/mdl.v113i5.13361

42. Tchir DR, Szafron ML. Occupational Health Needs and Predicted Well-Being in Office Workers Undergoing Web-Based Health Promotion Training: Cross-Sectional Study. *J Med Internet Res*. 2020;22(5):e14093. doi:10.2196/14093

43. Vahteristo A, Jylha V. Effects of User Participation in the Development of Health Information Systems on Their Evaluation Within Occupational Health Services. *Stud Health Technol Inform*. 2020;275(ck1, 9214582):207-211. doi:10.3233/SHTI200724

44. Volker D, Vlasveld MC, Anema JR, et al. Blended E-health module on return to work embedded in collaborative occupational health care for common mental disorders: design of a cluster randomized controlled trial. *Neuropsychiatr Dis Treat*. 2013;9(101240304):529-537. doi:10.2147/NDT.S43969

45. Volker D, Zijlstra-Vlasveld MC, Brouwers EPM, van der Feltz-Cornelis CM. Process Evaluation of a Blended Web-Based Intervention on Return to Work for Sick-Listed Employees with Common Mental Health Problems in the Occupational Health Setting. *J Occup Rehabil*. 2017;27(2):186-194. doi:10.1007/s10926-016-9643-4

46. Wattenberg EV, Bielicki JM, Suchomel AE, Sweet JT, Vold EM, Ramachandran G. Assessment of the Acute and Chronic Health Hazards of Hydraulic Fracturing Fluids. *J Occup Environ Hyg*. 2015;12(9):611-624. doi:10.1080/15459624.2015.1029612

47. Weichelt B, Bendixsen C, Keifer M. Farm Owners and Workers as Key Informants in User-Centered Occupational Health Prototype Development: A Stakeholder-Engaged Project. *J Med Internet Res*. 2019;21(1):e9711. doi:10.2196/jmir.9711

48. Werkmeister BJ, Haase AM, Fleming T, Officer TN. Experiences of the COVID-19 Lockdown and Telehealth in Aotearoa New Zealand: Lessons and Insights from Mental Health Clinicians. *Int J Environ Res Public Health*. 2023;20(6). doi:10.3390/ijerph20064791

49. White ND, Lenz TL, Skrabal MZ, et al. Comparison of cardiovascular risk calculation tools in pharmacy practice. *J Am Pharm Assoc JAPhA*. 2013;53(4):408-413. doi:10.1331/JAPhA.2013.12181

50. Yang L, Lu K, Diaz-Olivares JA, et al. Towards Smart Work Clothing for Automatic Risk Assessment of Physical Workload. *IEEE Access*. 2018;6:40059-40072. doi:10.1109/ACCESS.2018.2855719

51. Ye BJ, Kim JY, Suh C, et al. Development of a chatbot program for follow-up management of workers' general health examinations in korea: A pilot study. *Int J Environ Res Public Health*. 2021;18(4):1-14. doi:10.3390/ijerph18042170

52. Zamora-Ilarionov A, Rodriguez L. The use of eHealth to design a regional health promotion program in the workplace: Institute of Costa Rican Electricity case series. *mHealth*. 2020;6. doi:10.21037/mhealth-19-231

53. Guo SY, Ding LY, Luo HB, Jiang XY. A Big-Data-based platform of workers' behavior: Observations from the field. *Accid Anal Prev*. 2016;93(acs, 1254476):299-309. doi:10.1016/j.aap.2015.09.024

54. Manghisi VM, Fiorentino M, Boccaccio A, et al. A body tracking-based low-cost solution for monitoring workers' hygiene best practices during pandemics. *Sens Switz*. 2020;20(21):1-17. doi:10.3390/s20216149

55. Martin A, Kilpatrick M, Scott J, et al. Protecting the Mental Health of Small-to-Medium Enterprise Owners: A Randomized Control Trial Evaluating a Self-Administered Versus Telephone Supported Intervention. *J Occup Environ Med*. 2020;62(7):503-510. doi:10.1097/JOM.0000000000001882

56. Morris AS, Mackintosh KA, Owen N, Dempsey PC, Dunstan DW, McNarry MA. Rise and recharge: Exploring employee perceptions of and contextual factors influencing an individual-level e-health smartphone intervention to reduce office workers' sedentary time at work. *Int J Environ Res Public Health*. 2021;18(18). doi:10.3390/ijerph18189627

57. Peter L, Reindl R, Zauter S, Hillemacher T, Richter K. Effectiveness of an Online CBT-I Intervention and a Face-to-Face Treatment for Shift Work Sleep Disorder: A Comparison of Sleep Diary Data. *Int J Environ Res Public Health*. 2019;16(17). doi:10.3390/ijerph16173081

58. Atkins S, Reho T, Talola N, Sumanen M, Viljamaa M, Uitti J. Improved recording of work relatedness during patient consultations in occupational primary health care: a cluster randomized controlled trial using routine data. *Trials*. 2020;21(1):256. doi:10.1186/s13063-020-4168-8

59. Balk-Møller NC, Poulsen SK, Larsen TM. Effect of a nine-month web- and app-based workplace intervention to promote healthy lifestyle and weight loss for employees in the social welfare and health care sector: A randomized controlled trial. *J Med Internet Res*. 2017;19(4). doi:10.2196/jmir.6196

60. Boerema S, van Velsen L, Hermens H. An intervention study to assess potential effect and user experience of an mHealth intervention to reduce sedentary behaviour among older office workers. *BMJ Health Care Inform*. 2019;26(1). doi:10.1136/bmjhci-2019-100014

61. Bolier L, Ketelaar SM, Nieuwenhuijsen K, Smeets O, Gärtner FR, Sluiter JK. Workplace mental health promotion online to enhance well-being of nurses and allied health professionals: A cluster-randomized controlled trial. *Internet Interv*. 2014;1(4):196-204. doi:10.1016/j.invent.2014.10.002

62. Bort-Roig J, Chirveches-Perez E, Gine-Garriga M, et al. An mHealth Workplace-Based "Sit Less, Move More" Program: Impact on Employees' Sedentary and Physical Activity Patterns at Work and Away from Work. *Int J Environ Res Public Health*. 2020;17(23). doi:10.3390/ijerph17238844

63. Carr P, Kevitt F. Service user satisfaction with telemedicine in an occupational healthcare setting. *Occup Med Oxf Engl*. 2023;(a79, 9205857). doi:10.1093/occmed/kqad047

64. Chen W, Li T, Zou G, et al. Results of a cluster randomized controlled trial to promote the use of respiratory protective equipment among migrant workers exposed to organic solvents in small

and medium-sized enterprises. *Int J Environ Res Public Health*. 2019;16(17). doi:10.3390/ijerph16173187

65. Comtois KA, Mata-Greve F, Johnson M, Pullmann MD, Mosser B, Arean P. Effectiveness of Mental Health Apps for Distress During COVID-19 in US Unemployed and Essential Workers: Remote Pragmatic Randomized Clinical Trial. *JMIR MHealth UHealth*. 2022;10(11):e41689. doi:10.2196/41689

66. Cooley D, Pedersen S, Mainsbridge C. Assessment of the impact of a workplace intervention to reduce prolonged occupational sitting time. *Qual Health Res*. 2014;24(1):90-101. doi:10.1177/1049732313513503

67. Costa F, Janela D, Molinos M, et al. Impacts of Digital Care Programs for Musculoskeletal Conditions on Depression and Work Productivity: Longitudinal Cohort Study. *J Med Internet Res*. 2022;24(7):e38942. doi:10.2196/38942

68. De Kock JH, Latham HA, Cowden RG, et al. Brief Digital Interventions to Support the Psychological Well-being of NHS Staff During the COVID-19 Pandemic: 3-Arm Pilot Randomized Controlled Trial. *JMIR Ment Health*. 2022;9(4). doi:10.2196/34002

69. Ebert DD, Kahlke F, Buntrock C, et al. A health economic outcome evaluation of an internet-based mobile-supported stress management intervention for employees. *Scand J Work Environ Health*. 2018;44(2):171-182. doi:10.5271/sjweh.3691

70. Gayed A, Bryan BT, LaMontagne AD, et al. A Cluster Randomized Controlled Trial to Evaluate HeadCoach: An Online Mental Health Training Program for Workplace Managers. *J Occup Environ Med*. 2019;61(7):545-551. doi:10.1097/JOM.0000000000001597

71. Gilson ND, Pavey TG, Wright OR, et al. The impact of an m-Health financial incentives program on the physical activity and diet of Australian truck drivers. *BMC Public Health*. 2017;17(1):467. doi:10.1186/s12889-017-4380-y

72. Gwain GC, Amu H, Bain LE. Improving Employee Mental Health: A Health Facility-Based Study in the United States. *Front Public Health*. 2022;10:895048. doi:10.3389/fpubh.2022.895048

73. Haile C, Kirk A, Cogan N, Janssen X, Gibson AM, MacDonald B. Pilot Testing of a Nudge-Based Digital Intervention (Welbot) to Improve Sedentary Behaviour and Wellbeing in the Workplace. *Int J Environ Res Public Health*. 2020;17(16). doi:10.3390/ijerph17165763

74. Huang SJ, Hung WC, Shyu ML, Chou TR, Chang KC, Wai JP. Field Test of an m-Health Worksite Health Promotion Program to Increase Physical Activity in Taiwanese Employees: A Cluster-Randomized Controlled Trial. *Workplace Health Saf*. 2023;71(1):14-21. doi:10.1177/21650799221082304

75. Johnson KE, Alencar MK, Miller B, Gutierrez E, Dionicio P. Exploring Sex Differences in the Effectiveness of Telehealth-Based Health Coaching in Weight Management in an Employee Population. *Am J Health Promot AJHP*. 2021;35(2):262-265. doi:10.1177/0890117120943363

76. Judice PB, Hamilton MT, Sardinha LB, Silva AM. Randomized controlled pilot of an intervention to reduce and break-up overweight/obese adults' overall sitting-time. *Trials*. 2015;16(101263253):490. doi:10.1186/s13063-015-1015-4

77. Jukic T, Ihan A, Strojnik V, Stubljar D, Starc A. The effect of active occupational stress management on psychosocial and physiological wellbeing: a pilot study. *BMC Med Inform Decis Mak*. 2020;20(1). doi:10.1186/s12911-020-01347-z

78. Kempf K, Röhling M, Martin S, Schneider M. Telemedical coaching for weight loss in overweight employees: A three-armed randomised controlled trial. *BMJ Open*. 2019;9(4). doi:10.1136/bmjopen-2018-022242

79. Kouwenhoven-Pasmooij TA, Robroek SJW, Ling SW, et al. A blended web-based gaming intervention on changes in physical activity for overweight and obese employees: Influence and usage in an experimental pilot study. *JMIR Serious Games*. 2017;5(2). doi:10.2196/games.6421

80. Kouwenhoven-Pasmooij TA, Robroek SJW, Kraaijenhagen RA, et al. Effectiveness of the blended-care lifestyle intervention “PerfectFit”: a cluster randomised trial in employees at risk for cardiovascular diseases. *BMC Public Health*. 2018;18(1):766. doi:10.1186/s12889-018-5633-0

81. Lau EY, Faulkner G. Program implementation and effectiveness of a national workplace physical activity intervention: UPnGO with ParticipACTION. *Can J Public Health*. 2019;110(2):187-197. doi:10.17269/s41997-018-0170-2

82. Lavaysse LM, Imrisek SD, Lee M, et al. One Drop Improves Productivity for Workers With Type 2 Diabetes: One Drop for Workers With Type 2 Diabetes. *J Occup Environ Med*. 2022;64(8):E452-E458. doi:10.1097/JOM.0000000000002577

83. Lee SH, Ha Y, Jung M, Yang S, Kang WS. The Effects of a Mobile Wellness Intervention with Fitbit Use and Goal Setting for Workers. *Telemed E-Health*. 2019;25(11):1115-1122. doi:10.1089/tmj.2018.0185

84. Lennefer T, Reis D, Lopper E, Hoppe A. A step away from impaired well-being: a latent growth curve analysis of an intervention with activity trackers among employees. *Eur J Work Organ Psychol*. 2020;29(5):664-677. doi:10.1080/1359432X.2020.1760247

85. Lokman S, Volker D, Zijlstra-Vlasveld MC, et al. Return-to-work intervention versus usual care for sick-listed employees: health-economic investment appraisal alongside a cluster randomised trial. *BMJ Open*. 2017;7(10):e016348. doi:10.1136/bmjopen-2017-016348

86. MacDonald B, Gibson AM, Janssen X, Kirk A. A Mixed Methods Evaluation of a Digital Intervention to Improve Sedentary Behaviour Across Multiple Workplace Settings. *Int J Environ Res Public Health*. 2020;17(12). doi:10.3390/ijerph17124538

87. Mainsbridge CP, Cooley PD, Fraser SP, Pedersen SJ. The effect of an e-health intervention designed to reduce prolonged occupational sitting on mean arterial pressure. *J Occup Environ Med*. 2014;56(11):1189-1194. doi:10.1097/JOM.0000000000000243

88. Mainsbridge C, Ahuja K, Williams A, Bird ML, Cooley D, Pedersen SJ. Blood pressure response to interrupting workplace sitting time with non-exercise physical activity results of a 12-month cohort study. *J Occup Environ Med*. 2018;60(9):769-774. doi:10.1097/JOM.0000000000001377

89. Maylor BD, Edwardson CL, Zakrzewski-Fruer JK, Champion RB, Bailey DP. Efficacy of a Multicomponent Intervention to Reduce Workplace Sitting Time in Office Workers: A Cluster Randomized Controlled Trial. *J Occup Environ Med*. 2018;60(9):787-795. doi:10.1097/JOM.0000000000001366

90. Meyer D, Jayawardana MW, Muir SD, Ho DYT, Sackett O. Promoting Psychological Well-Being at Work by Reducing Stress and Improving Sleep: Mixed-Methods Analysis. *J Med Internet Res*. 2018;20(10):e267. doi:10.2196/jmir.9058

91. Michelsen C, Kjellgren A. The Effectiveness of Web-Based Psychotherapy to Treat and Prevent Burnout: Controlled Trial. *JMIR Form Res*. 2022;6(8):e39129. doi:10.2196/39129

92. Morris AS, Mackintosh KA, Dunstan D, et al. Rise and recharge: Effects on activity outcomes of an e-health smartphone intervention to reduce office workers' sitting time. *Int J Environ Res Public Health*. 2020;17(24):1-18. doi:10.3390/ijerph17249300

93. Muniswamy P, Gorhe V, Parashivakumar L, Chandrasekaran B. Short-term effects of a social media-based intervention on the physical and mental health of remotely working young software professionals: A randomised controlled trial. *Appl Psychol Health Well-Being*. 2022;14(2):537-554. doi:10.1111/aphw.12318

94. Nagata T, Aoyagi SS, Takahashi M, Nagata M, Mori K. Effects of Feedback From Self-Monitoring Devices on Lifestyle Changes in Workers with Diabetes: 3-Month Randomized Controlled Pilot Trial. *JMIR Form Res*. 2022;6(8). doi:10.2196/23261

95. Notenbomer A, Roelen C, Groothoff J, Van Rhenen W, Bültmann U. Effect of an eHealth intervention to reduce sickness absence frequency among employees with frequent sickness absence: Randomized controlled trial. *J Med Internet Res*. 2018;20(10). doi:10.2196/10821

96. Nundy S, Dick JJ, Chou CH, Nocon RS, Chin MH, Peek ME. Mobile phone diabetes project led to improved glycemic control and net savings for Chicago plan participants. *Health Aff Proj Hope*. 2014;33(2):265-272. doi:10.1377/hlthaff.2013.0589

97. Park JH, Jung SE, Ha DJ, et al. The effectiveness of e-healthcare interventions for mental health of nurses: A PRISMA-compliant systematic review of randomized controlled trials. *Med U S*. 2022;101(25):E29125. doi:10.1097/MD.0000000000029125

98. Pedersen SJ, Cooley PD, Mainsbridge C. An e-health intervention designed to increase workday energy expenditure by reducing prolonged occupational sitting habits. *Work*. 2014;49(2):289-295. doi:10.3233/WOR-131644

99. Ryu H, Jung J, Moon J. Effectiveness of a Mobile Health Management Program With a Challenge Strategy for Improving the Cardiovascular Health of Workers. *J Occup Environ Med*. 2021;63(3):e132-e137. doi:10.1097/JOM.0000000000002130

100. Röhling M, Martin K, Ellinger S, Schreiber M, Martin S, Kempf K. Weight reduction by the low-insulin-method— a randomized controlled trial. *Nutrients*. 2020;12(10):1-17. doi:10.3390/nu12103004

101. Sasaki N, Imamura K, Tran TTT, et al. Effects of Smartphone-Based Stress Management on Improving Work Engagement Among Nurses in Vietnam: Secondary Analysis of a Three-Arm Randomized Controlled Trial. *J Med Internet Res*. 2021;23(2):e20445. doi:10.2196/20445

102. Simons LPA, Hafkamp MPJ, Van Bodegom D, Dumaij A, Jonker CM. Improving employee health; Lessons from an RCT. *Int J Netw Virtual Organ*. 2017;17(4):341-353. doi:10.1504/IJNVO.2017.088485

103. Thogersen-Ntoumani C, Quested E, Smith BS, et al. Feasibility and preliminary effects of a peer-led motivationally-embellished workplace walking intervention: A pilot cluster randomized

trial (the START trial). *Contemp Clin Trials*. 2020;91(101242342):105969. doi:10.1016/j.cct.2020.105969

104. Umanodan R, Shimazu A, Minami M, Kawakami N. Effects of computer-based stress management training on psychological well-being and work performance in japanese employees: a cluster randomized controlled trial. *Ind Health*. 2014;52(6):480-491.

105. Van Schaaijk A, Nieuwenhuijsen K, Frings-Dresen M. Work ability and vitality in coach drivers: An rct to study the effectiveness of a self-management intervention during the peak season. *Int J Environ Res Public Health*. 2019;16(12). doi:10.3390/ijerph16122214

106. Volker D, Zijlstra-Vlasveld MC, Anema JR, et al. Effectiveness of a blended web-based intervention on return to work for sick-listed employees with common mental disorders: Results of a cluster randomized controlled trial. *J Med Internet Res*. 2015;17(5). doi:10.2196/jmir.4097

107. Widmer RJ, Allison TG, Keane B, Dallas A, Lerman LO, Lerman A. Using an online, personalized program reduces cardiovascular risk factor profiles in a motivated, adherent population of participants. *Am Heart J*. 2014;167(1):93-100. doi:10.1016/j.ahj.2013.09.019

108. Widmer RJ, Allison TG, Keane B, et al. Workplace digital health is associated with improved cardiovascular risk factors in a frequency-dependent fashion: A large prospective observational cohort study. *PLoS ONE*. 2016;11(4). doi:10.1371/journal.pone.0152657

109. Willman AS. Evaluation of eConsult use by Defence Primary Healthcare primary care clinicians using a mixed-method approach. *BMJ Mil Health*. 2023;169(e1):e39-e43. doi:10.1136/bmjmilitary-2020-001660

110. Wipfli B, Hanson G, Anger K, et al. Process Evaluation of a Mobile Weight Loss Intervention for Truck Drivers. *Saf Health Work*. 2019;10(1):95-102. doi:10.1016/j.shaw.2018.08.002
