# Supplementary material for: Impact of Digital Interventions in Occupational Health Care: A Systematic Review
Source: Mayo Clin Proc Digit Health. 2025 Mar 18;3(2):100216. doi: 10.1016/j.mcpdig.2025.100216 (PMC12190893; doi:10.1016/j.mcpdig.2025.100216)
Supplement: Supplemental Material [file mmc3.pdf]

THE LANCET
Digital Health

Author statements

Please insert the relevant text under the subheadings below. A completed form must be signed by all authors. Please note that we will accept hand-signed and electronic (typewritten) signatures. Please complete multiple forms if necessary, and upload the signed copy with your submission, scan and email to: digitalhealth@lancet.com.

Manuscript title: Impact of digital interventions in occupational healthcare - a systematic review

Corresponding author: Mirjam Jern-Matintupa

Article type: Systematic review

I irrevocably authorise and grant my full consent to the corresponding author of the manuscript to: (1) enter into an exclusive publishing agreement with Elsevier on on my behalf (or, if the article is to be published under a CC BY license, a non-exclusive publishing agreement), in the relevant form set out at www.elsevier.com/copyright; and (2) unless I am a US government employee, to transfer my copyright or grant an exclusive license of rights (or, for CC BY articles, a non-exclusive license of rights) to Elsevier as part of that publishing agreement, effective on acceptance of the article for publication. If the article is a work made for hire, I am authorized to confirm this on behalf of my employer. I agree that the copyright status selected by the corresponding author for the article if it is accepted for publication shall apply and that this agreement is subject to the governing law of the country in which the journal owner is located.

Does your manuscript have a reference number? No ◉ Yes ○ If yes, enter number here:

Does your manuscript have a handling editor? No ◉ Yes ○ If yes, enter name here:

Authors' contributions

Please insert here the contribution each author made to the manuscript—eg, literature search, figures, study design, data collection, data analysis, data interpretation, writing etc. You may also find the CRediT taxonomy helpful. See http://credit.niso.org/ for more information on 14 roles that can be attributed to authors: conceptualisation, data curation, formal analysis, funding acquisition, investigation, methodology, project administration, resources, software, supervision, validation, visualisation, writing – original draft, and writing – review & editing. If all authors contributed equally, please state this. The information provided here must match the contributors' statement in the manuscript. We require that more than one author has directly accessed and verified the underlying data reported in the manuscript. For research articles that are the result of an academic and commercial partnership, at least one of the authors named as having accessed and verified data must be from the academic team.

The search strategy and study design was designed by Mirjam Jern-Matintupa, supervised and reviewed by Merja K Laine and Anita Riipinen.
The screenings of articles and data collection was coducted by all authors.
Data analysis condcted by Mirjam Jern-Matintupa and supervised and reviewed by Merja K Laine and Anita Riipinen
Data interpretation was conducted by all authors.
Writing of original draft was done by Mirjam Jern-Matintupa
Writing, reviewing and editing of the final manuscript was conducted by all athours
All authors confirm that they had full access to all the data in the study and accept responsibility to submit for publication

THE LANCET
Digital Health

Role of the funding source
Please disclose any funding sources and their role, if any, in the writing of the manuscript or the decision to submit it for publication. Examples of involvement include: data collection, analysis, or interpretation; trial design; patient recruitment; or any aspect pertinent to the study. Please also comment whether you have been paid to write this article by a pharmaceutical company or other agency. I The information provided here must match the role of the funding source statement in the manuscript. If you are the corresponding author, please state that authors were not precluded from accessing data in the study, and they accept responsibility to submit for publication.

Mirjam Jern-Matintupa received funding for her doctoral studies from Finska Läkare Sällskapet in 2023 and 2024 that has been used in part to fund her work on this study.
The funder of the study had no role in study design, data collection, data analysis, data interpretation or writing of the report.
The authors were not precluded from accessing data in the study, and they accept responsibility to submit for publication

Conflicts of interest
Please complete the ICMJE conflict of interest form, which is available at https://www.thelancet.com/for-authors/forms?section=icmje-coi. Please ensure that a conflict of interest statement is included at the end of the manuscript, which matches what is declared on the ICMJE conflict of interest form.

Patient consent (if applicable) - completion of this section is mandatory for Case Reports, Clinical Pictures, and Adverse Drug Reactions.
Please sign below to confirm that all necessary consents required by applicable law from any relevant patient, research participant, and/or other individual whose information is included in the article have been obtained in writing. **The signed consent form(s) should be retained by the corresponding author and NOT sent to *The Lancet Digital Health*.**

I agree with: the plan to submit to *The Lancet Digital Health*; the contents of the manuscript; the statements on data access; to being listed as an author; and to the conflicts of interest statement as summarised.

Title and name: Mrs Mirjam Jern-Matintupa	Highest degree: ML	Signature: Mirjam Jern-Matintupa	Date: 25.6
Title and name: Ass Prof Merja Laine	Highest degree: MD	Signature:	Date:
Title and name: Mrs Anita Riipinen	Highest degree: MD	Signature:	Date:
Title and name:	Highest degree:	Signature:	Date:
Title and name:	Highest degree:	Signature:	Date:
Title and name:	Highest degree:	Signature:	Date:
Title and name:	Highest degree:	Signature:	Date:
Title and name:	Highest degree:	Signature:	Date:
Title and name:	Highest degree:	Signature:	Date:
Title and name:	Highest degree:	Signature:	Date:

Corresponding author declaration

I Mirjam Jern-Matintupa [Signature], the corresponding author of this manuscript, certify that the contributors' and conflicts of interest statements included in this paper are correct and have been approved by all co-authors.
